# The effect of Link N on differentiation of human bone marrow-derived mesenchymal stem cells

**DOI:** 10.1186/ar4113

**Published:** 2012-12-10

**Authors:** John Antoniou, Hong Tian Wang, Abdulrahman M Alaseem, Lisbet Haglund, Peter J Roughley, Fackson Mwale

**Affiliations:** 1 Division of Orthopaedic Surgery, McGill University, 1650 Cedar Avenue, Montreal, QC, H3G 1A4, Canada; 2 Lady Davis Institute for Medical Research, SMBD-Jewish General Hospital, 3755 Chemin de la Cote Ste-Catherine, Montreal, QC, H3T 1E2, Canada; 3 Orthopaedic Research Laboratory, Royal Victoria Hospital, McGill University, 687 Pine Avenue West Montreal, QC, H3A 1A1, Canada; 4 Genetics Unit, Shriners Hospitals for Children, 1529 Cedar Avenue, Montreal, QC, H3G 1A6, Canada

## Abstract

**Introduction:**

We previously showed that Link N can stimulate extracellular matrix biosynthesis by intervertebral disc (IVD) cells, both *in vitro *and *in vivo*, and is therefore a potential stimulator of IVD repair. The purpose of the present study was to determine how Link N may influence human mesenchymal stem cell (MSC) differentiation, as a prelude to using Link N and MSC supplementation in unison for optimal repair of the degenerated disc.

**Methods:**

MSCs isolated from the bone marrow of three osteoarthritis patients were cultured in chondrogenic or osteogenic differentiation medium without or with Link N for 21 days. Chondrogenic differentiation was monitored by proteoglycan staining and quantitation by using Alcian blue, and osteogenic differentiation was monitored by mineral staining and quantitation by using Alzarin red S. In addition, proteoglycan secretion was monitored with the sulfated glycosaminoglycan (GAG) content of the culture medium, and changes in gene expression were analyzed with real-time reverse transcription (RT) PCR.

**Results:**

Link N alone did not promote MSC chondrogenesis. However, after MSCs were supplemented with Link N in chondrogenic differentiation medium, the quantity of GAG secreted into the culture medium, as well as aggrecan, *COL2A1*, and *SOX9 *gene expression, increased significantly. The gene expression of *COL10A1 *and osteocalcin (*OC*) were downregulated significantly. When MSCs were cultured in osteogenic differentiation medium, Link N supplementation led to a significant decrease in mineral deposition, and alkaline phosphatase (*ALP*), *OC*, and *RUNX2 *gene expression.

**Conclusions:**

Link N can enhance chondrogenic differentiation and downregulate hypertrophic and osteogenic differentiation of human MSCs. Therefore, in principle, Link N could be used to optimize MSC-mediated repair of the degenerated disc.

## Introduction

Intervertebral disc (IVD) degeneration plays a major role in the etiology of low-back pain (LBP), which can significantly affect more than half of the population at some point during their lives [1-3]. Disc degeneration usually is associated with increased breakdown of matrix, decreased synthesis of aggrecan and collagen, cell loss through apoptosis, and cluster formation by surviving cells [4-7]. It is evident that vascular ingrowth can occur into the degenerated IVD, and that in painful degenerated IVDs, the vessels are accompanied by nociceptive nerves [1]. Thus, reversing the degeneration process and repairing the degenerated IVDs is important for LBP therapy.

Disc repair may be enhanced by cell supplementation to maximize extracellular matrix production. The source of cells for disc repair is not immediately apparent, as no benign site is known for harvesting authentic autologous disc cells, and it is preferable to avoid the use of allogeneic donor disc cells. Although the avascular nature of the disc may make the nucleus pulposus (NP) an immunologically privileged site, and therefore make the use of allogeneic cells a tempting proposition, the risk of transferring infectious agents remains real. Thus, we must generate disc cells from another autologous source.

One possibility is to use mesenchymal stem cells (MSCs). MSCs are multipotent stem cells that can be isolated, expanded, and stimulated to differentiate into a variety of cells, including osteoblasts, chondrocytes, myocytes, adipocytes, and beta pancreatic islets cells [8-11]. Recent studies have shown that MSCs can be used in biologic repair of cartilage or IVD lesions, because of the bioactive factors secreted by MSCs and the proliferation of chondrocytes differentiated from MSCs [12-18]. MSCs can be injected directly, or together with a scaffold, into the degenerated disc, where they can differentiate into disc cells, produce extracellular matrix (ECM), and reestablish healthy disc function [19-22]. It is well known that growth factors, such as bone morphogenetic proteins (BMPs) and transforming growth factor-β (TGF-β) can be applied directly for tissue regeneration. Injecting MSCs together with growth factors into IVDs can bring added benefits to the repair process, considering that growth factors and MSCs can improve tissue repair individually, and growth factors can stimulate and accelerate the differentiation of MSCs to chondrocytes.

Hyaline cartilage and healthy NP possess similar macromolecules in their extracellular matrix [23-25], although some differences in the structure of proteoglycans in cartilage and the NP have been observed [26]. The production of an extracellular matrix with a high proteoglycan-to-collagen ratio can distinguish NP cells from chondrocytes and could help in identifying an NP-like phenotype *in vivo*, as opposed to a chondrocyte when MSCs are induced to differentiate for tissue engineering of a disc [27].

As an economical alternative to growth factors, it may be possible to use Link N together with MSCs for tissue regeneration. Link N (DHLSDNYTLDHDRAIH) is the N-terminal peptide of link protein, a glycoprotein that stabilizes the non-covalent interaction between an aggrecan G1 domain and hyaluronate. Link N can stimulate the synthesis of collagen in human articular cartilage and bovine IVD cells *in vitro *[28-30], as well as in the IVDs of rabbits *in vivo *[31]. Our previous results showed that Link N can also decrease the expression of type X collagen, a marker of chondrocyte hypertrophy [32], and stimulate the expression of type II collagen, a marker of cartilage and disc ECM [33], in human mesenchymal stem cells [34]. Thus, Link N has the potential to be used together with MSCs in promoting the formation of the type of ECM necessary for IVD repair. However, to be useful for this purpose, it is essential that Link N does not interfere with MSC chondrogenesis.

The purpose of this research was to determine the effects of Link N on human MSCs cultured in chondrogenic and osteogenic differentiation media to determine how Link N affects these processes.

## Materials and methods

### Source and isolation of stem cells

MSCs were obtained from aspirates of the intramedullary canal of three osteoarthritis patients (aged 40 to 60 years) undergoing total hip replacement, with a protocol approved by the Research Ethics Committee of the Jewish General Hospital (Montreal, QC, Canada). We had all necessary consent from any patients involved in the study, including consent to participate in the study and consent to publish. Bone marrow aspirates were processed as previously described [34]. In brief, each aspirate was diluted 1:1 (vol/vol) with Dulbecco Modified Eagle Medium (DMEM) containing 4.5 g/L glucose, L-glutamine, and sodium pyruvate (Wisent Inc., St-Bruno, QC, Canada) and then layered over Ficoll (Ficoll-Paque Plus; GE Healthcare Bio-Sciences, Baie-d'Urfé, QC, Canada). After centrifugation at 900 *g *for 30 minutes, the mononuclear cell layer was removed from the interface, washed with DMEM, and resuspended in DMEM supplemented with 10% fetal bovine serum (FBS; Hyclone, Logan, UT, USA), 100 units/ml penicillin, and 100 μg/ml streptomycin. The cells were plated in culture dishes and incubated at 37**°**C in a humidified atmosphere with 5% CO_2_. After 48 hours, nonadherent cells were washed off, and the adherent cells were thoroughly washed twice with DMEM. All cells were expanded in DMEM supplemented with 10% FBS, 100 units/ml penicillin, 100 μg/ml streptomycin, and were used within four passages.

### Cell culture

In every well of a 24-well plate, about 4,000 MSCs were plated and cultured in expansion medium, high glucose DMEM with 10% FBS, 100 units/ml penicillin, and 100 μg/ml streptomycin. Floating cells were removed after allowing MSCs to attach overnight, and then the attached MSCs were cultured in 1 ml of the same medium for 3 days. The cells were washed with phosphate-buffered saline (PBS) 3 times, and then were cultured in chondrogenic or osteogenic differentiation medium. Link N was dissolved in the differentiation media at a final concentration of 0.1 μg/ml or 1.0 μg/ml. Differentiation medium without Link N was used as a control. The medium was changed every 3 days. The used chondrogenic differentiation media were stored at -20°C for GAG analysis.

For gene expression analysis, 5 × 10^5 ^MSCs were plated on 35 × 10-mm dishes (Sarstedt, Quebec, QC, Canada). After the cells were cultured in expansion medium (see earlier) for 3 days, they were cultured in chondrogenic or osteogenic differentiation medium with the same conditions as earlier.

To compare the direct effects of Link N and TGF-β3 on MSC chondrogenesis, MSCs were cultured in serum-free medium, serum-free medium with 1 μg/ml Link N, or serum-free medium with 10 ng/ml TGF-β3 for 21 days. Serum-free medium was prepared with high-glucose DMEM with 1 × ITS+1 premix (Sigma-Aldrich, Ontario, QC, Canada), 100 n*M *dexamethasone, 50 μg/ml ascorbic acid-2-phosphate, 40 μg/ml proline, 100 units/ml penicillin, and 100 μg/ml streptomycin.

### Chondrogenic differentiation and proteoglycan analysis

Chondrogenic differentiation medium containing TGF-β was from Invitrogen (Burlington, ON, Canada). Chondrogenic differentiation was monitored by proteoglycan accumulation by using Alcian blue staining [35]. In brief, after the cells were cultured for 21 days, the culture medium was removed, the cells were rinsed gently with PBS twice, and then fixed with -20°C methanol for 30 minutes. After rinsing with PBS twice, the wells were stained with Alcian blue in 0.1N HCl for 30 minutes. The wells were then rinsed with distilled water 3 times; and images of stained wells were captured. To quantify proteoglycan, the matrix-associated dye was extracted with 6 *M *guanidine-HCl (Sigma-Aldrich, Oakville, ON, Canada; 200 μl/well) and measured at 620 nm.

### Osteogenic differentiation and mineralization analysis

The osteogenic differentiation medium was prepared with high-glucose DMEM containing 10% FBS, 0.1 μ*M *dexamethasone, 10 m*M *β-glycerophosphate, 50 μ*M *L-ascorbic acid, 100 units/ml penicillin, and 100 μg/ml streptomycin. Osteogenic differentiation was monitored by mineral deposition by using alizarin red S staining [36]. In brief, after the cells were cultured for 21 days, the medium was removed, and the cells were rinsed once with distilled water. The cells were then fixed with 70% ethanol (stored at -20°C) for 30 minutes. After fixation, the wells were rinsed twice with distilled water, and the cells were stained with 2% alizarin red S (pH 4.2) for 30 minutes. Finally, the wells were rinsed 3 times with distilled water, and images of stained wells were captured. To quantify the matrix mineralization, alizarin red S was extracted with 100 m*M *cetylpyridinium chloride (Sigma-Aldrich; 1 ml/well) and measured at 570 nm.

### GAG and DNA analysis

The sulfated glycosaminoglycan (GAG) content of the culture medium was detected by using a 96-well round-bottom plate [37]. To 20 μl culture medium in every well, 180 μl of 16 mg/L 1,9-dimethylmethylene blue (DMMB; Sigma-Aldrich) solution was added. The absorbance of the solution was monitored immediately at 535 nm. Chondroitin 6-sulfate from shark cartilage was used as a standard. DNA was measured on days 3 and 21 by using a Quant-iT dsDNA High-Sensitivity Assay Kit (Invitrogen, Burlington, ON, Canada), following the manufacturer's instructions. In brief, 20 μl of DNA solution was mixed with 200 μl of working solution in each well of a 96-well plate. Fluorescence measurements were taken with an excitation wavelength of 480 nm and emission wavelength of 530 nm. A standard curve was obtained from λ DNA.

### Total RNA isolation

Cells were harvested at days 7, 14, and 21 for gene-expression studies. Total RNA was extracted from MSCs by using Trizol (Invitrogen), following the manufacturer's instructions. After centrifugation for 15 minutes at 12,000 *g *at 4**°**C, the aqueous phase was precipitated with 1 volume of isopropanol, incubated for 45 minutes at -20**°**C, and centrifuged again for 15 minutes at 12,000 *g *at 4**°**C. The resulting RNA pellet was washed with 75% ethanol, centrifuged, and air-dried. The pellets were dissolved in 50 μl diethylpyrocarbonate (DEPC)-treated distilled water and assayed for RNA concentration, by measuring A_260_, and purity, by calculating the A_260_/A_280 _ratio.

### Reverse transcription

One microgram total RNA isolated from the cells was digested with DNase I (Invitrogen). Then, 1 μg RNA was mixed with random primers (final concentration, 0.15 μg/μl), dNTP mixture (final concentration 0.5 m*M*), and DEPC-treated distilled water to a total volume of 12 μl. Following the instructions of the reagent supplier (Invitrogen), the solution was incubated at 65°C for 5 minutes, and then it was mixed with reaction buffer, DTT, RNaseOUT, and Superscript II reverse transcriptase with a final volume of 20 μl. The solution was incubated at 45°C for 50 minutes and then at 70°C for 15 minutes to inactivate the reverse transcriptase.

### Real-time PCR

For LightCycler real-time PCR, a master mix of the following reaction components was prepared: 10 μl SYBER PCR master mix (×1) (Qiagen, Mississauga, ON, Canada), 8 μl distilled water, 0.5 μl forward primer (0.25 μ*M*), and 0.5 μl reverse primer (0.25 μ*M*). The nucleotide sequences of the primers are listed in Table 1. To each 19 μl master mix, 1 μl of cDNA was added as the PCR template. The PCR conditions included one cycle of initial activation (95°C for 15 minutes, 20°C/s ramp rate), 45 cycles of amplification and quantification (94°C for 15 seconds, 57°C for 30 seconds, 72°C for 30 seconds), one cycle of melting-curve determination (65°C to 95°C with heating rate of 0.1°C per second, with a continuous fluorescence measurement), and final cooling to 4°C.

**Table 1:** Primer sequences

Gene	Sequence	Size
*ACAN*	Forward (6708-6727): TGA GTC CTC AAG CCT CCT GT	185 bp
	Reverse (6873-6892): CCT CTG TCT CCT TGC AGG TC	

*ALP*	Forward (1397-1416): CCA CGT CTT CAC ATT TGG TG	196 bp
	Reverse(1573-1592): AGA CTG CGC CTG GTA GTT GT	

*COL10A1*	Forward (1670-1690): AAT GCC TGT GTC TGC TTT TAC	130 bp
	Reverse (1779-1799): ACA AGT AAA GAT TCC AGT CCT	

*COL2A1*	Forward (459-478): ATT TCA AGG CAA TCC TGG TG	218 bp
	Reverse (657-676): GGC CTG GAT AAC CTC TGT GA	

*GAPDH*	Forward (113-133): TGA AGG TCG GAG TCA ACG GAT	181 bp
	Reverse (273-293): TTC TCA GCC TTG ACG GTG CCA	

*OC*	Forward (20-39): TGA GAG CCC TCA CAC TCC TC	151 bp
	Reverse (170-151): CGC CTG GGT CTC TTC ACT AC	

*RUNX 2*	Forward (1312-1331): CAG ACC AGC AGC ACT CCA TA	178 bp
	Reverse (1470-1489): CAG CGT CAA CAC CAT CAT TC	

*SOX9*	Forward (19-38): TTC ATG AAG ATG ACC GAC GA	175 bp
	Reverse (174-193): CGC TCT CCT TCT TCA GAT CG	

The crossing points (CPs) were determined by the LightCycler software 3.3 (Roche Diagnostics, Indianapolis, IN, USA) and were measured at constant fluorescence level. The ratio of gene expression relative to *GAPDH *as the reference gene was determined by the following equation [38]:

$$\text{Relative ratio}=\frac{2^{\text{Δ}\text{C}{\text{P}}_{\text{target}}\left(\text{control}-\text{sample}\right)}}{2^{\text{Δ}\text{C}{\text{P}}_{\text{reference}}\left(\text{control}-\text{sample}\right)}}$$

### Statistical analysis

Statistical analysis was performed by using analysis of variance followed by Fisher protected least significant difference *post hoc *test by using Statview (SAS Institute Inc., Cary, NC, USA). Results are presented as the mean ± standard deviation (SD) of three independent experiments with cells from three different donors. Differences were considered statistically significant with *P *< 0.05.

## Results

**Figure 1:**
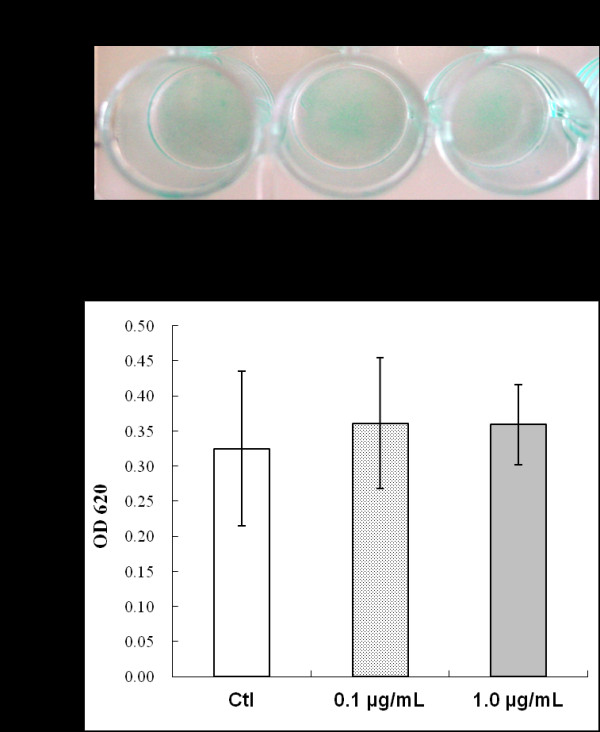
**Effect of Link N on proteoglycan deposition by mesenchymal stem cells (MSCs) in chondrogenic differentiation medium**. **(A) **Alcian blue staining of proteoglycan in extracellular matrix after MSCs were cultured in chondrogenic differentiation medium without (control) or with Link N for 21 days. **(B) **The absorbance value of the solubilized Alcian blue at 620 nm. The results are shown as mean ± SD of three independent experiments with MSCs from three different donors.

Analysis of MSCs cultured in chondrogenic differentiation medium with or without Link N demonstrated differentiation into a chondrogenic lineage by Alcian blue staining (Figure 1A). When Alcian blue was extracted from the stained cultures (Figure 1B), no significant differences in proteoglycan deposition were found between control cells and cells cultured with Link N.

**Figure 2:**
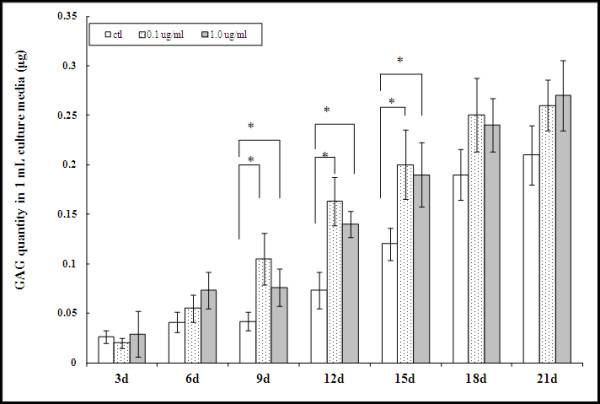
**Glycosaminoglycan (GAG) secretion into 1-ml culture medium over the 3-day period preceding medium collection when mesenchymal stem cells (MSCs) were cultured in chondrogenic differentiation medium in the absence (control) or presence of Link N**. The results are shown as mean ± SD of three independent experiments with MSCs from three different donors. *P < 0.05 versus control.

Proteoglycan production during chondrogenic differentiation can also be monitored with GAG analysis, and this was used to assess the effect of Link N on MSC differentiation. After MSCs were cultured in chondrogenic differentiation medium with Link N for 9, 12, and 15 days, the quantity of GAG secreted into the culture medium was significantly higher than that in control medium (Figure 2). This difference was not observed at days 3, 6, 18, and 21, although a tendency to increased GAG synthesis with Link N supplementation was observed on days 6, 18, and 21. Because the DNA content of the cultures did not change significantly between days 3 and 21 of culture, the enhanced GAG synthesis is likely the result of increased production by each cell rather than a consequence of having more cells because of cell proliferation.

**Figure 3:**
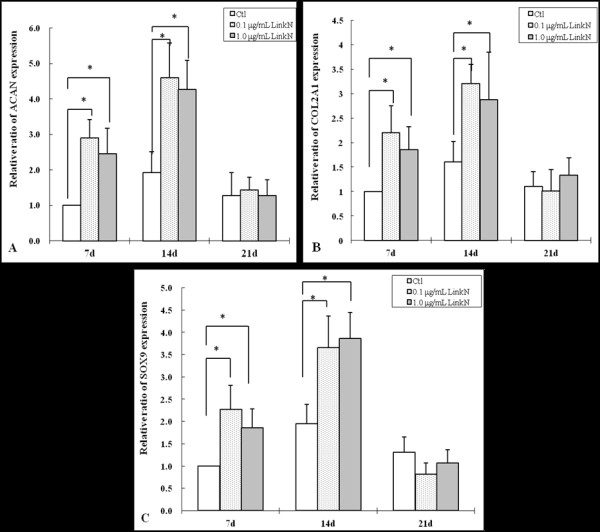
**Relative ratio of *ACAN *(A), *COL2A1 *(B), and *SOX9 *(C) gene expression in mesenchymal stem cells (MSCs) cultured in chondrogenic differentiation medium without (control) or with 0.1 or 1.0 μg/ml Link N for 7, 14, and 21 days**. The results are shown as mean ± SD of three independent experiments with MSCs from three different donors. *P < 0.05 versus control.

Aggrecan (*ACAN*) and *COL2A1 *are two important genes that define the chondrocyte phenotype [4,5], and *SOX9 *is an important transcription regulator of chondrogenesis [39,40]. The effect of Link N on the expression of these genes was therefore assessed. After the cells were cultured for 7 days in chondrogenic differentiation medium, *ACAN *expression with 0.1 μg/ml or 1.0 μg/ml Link N increased significantly compared with controls (*P *= 0.001 and 0.002, respectively) (Figure 3A). The increase was also observed after 14 days (*P *= 0.003 for 0.1 μg/ml Link N; *P *= 0.004 for 1.0 μg/ml Link N). Similarly, *COL2A1 *expression was also increased compared with control cells at both day 7 (*P *= 0.003 for 0.1 μg/ml Link N; *P *= 0.005 for 1.0 μg/ml Link N) and day 14 (*P *= 0.003 for 0.1 μg/ml Link N, *P *= 0.002 for 1.0 μg/ml Link N) (Figure 3B). Compared with control cells, the expression of *SOX9 *was increased at day 7 (*P *= 0.017 for 0.1 μg/ml Link N; *P *= 0.027 for 1.0 μg/ml Link N) and day 14 (*P *= 0.024 for 0.1 μg/ml Link N; *P *= 0.011 for 1.0 μg/ml Link N) (Figure 3C). For the expression of all three genes, no significant difference was observed between the two concentrations of Link N. Thus Link N appeared to promote the chondrogenic differentiation of MSCs.

**Figure 4:**
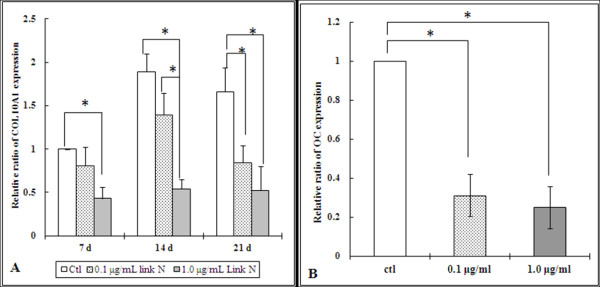
**Relative ratio of *COL10A1 *(A) and *OC *(B) gene expression in mesenchymal stem cells (MSCs) cultured in chondrogenic differentiation medium without (control) or with 0.1 or 1.0 μg/ml Link N**. The expression of *COL10A1 *was detected after the cells were cultured for 7, 14, or 21 days. The expression of *OC *was detected after the cells were cultured for 21 days. The results are shown as mean ± SD of three independent experiments with MSCs from three different donors. *P < 0.05 versus control.

*COL10A1 *is a marker gene for hypertrophic chondrocyte differentiation, a process that is undesirable for effective disc or cartilage repair [41,42]. When MSCs were cultured in chondrogenic differentiation medium with 1.0 μg/ml Link N for 7 (*P *= 0.002), 14 (*P *= 0.001) and 21 days (*P *= 0.007), gene expression was downregulated significantly (Figure 4A). When MSCs were cultured in chondrogenic differentiation medium with 0.1 μg/ml Link N for 7 and 14 days, no significant difference of *COL10A1 *expression was observed compared with the control cells. However, a significant difference was observed at day 21 (*P *= 0.015). The difference between cells cultured in different concentrations of Link N was significant at day 7 (*P *= 0.006). Thus, Link N appears to reduce hypertrophic differentiation by the MSCs.

Because another major problem with MSC-based chondrogenesis is their osteogenic differentiation, we next explored the effect of Link N on osteocalcin (*OC*), an important osteogenic marker [43,44]. When MSCs were cultured in chondrogenic differentiation medium with 0.1 μg/ml and 1.0 μg/ml Link N for 21 days, the expression of *OC *was significantly downregulated (*P *< 0.001) (Figure 4B).

**Figure 5:**
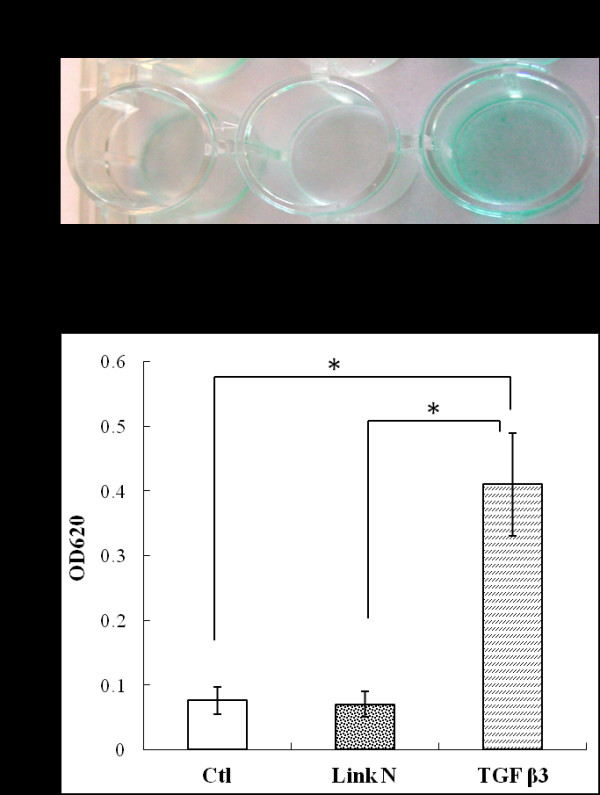
**Effect of Link N on proteoglycan deposition by mesenchymal stem cells (MSCs) in serum-free medium**. **(A) **Alcian blue staining of proteoglycan in extracellular matrix after MSCs were cultured in serum-free medium devoid of TGF-β (control), serum-free medium with 1 μg/ml Link N, and serum-free medium with 10 ng/ml TGF-β3 for 21 days. **(B) **The absorbance value of the solubilized Alcian blue at 620 nm. The results are shown as mean ± SD of three independent experiments with MSCs from three different donors.

To determine whether Link N can directly stimulate chondrogenesis, MSCs were cultured in serum-free medium without (control) or with either Link N or TGF-β3. Proteoglycan deposition in the accumulated ECM was visualized by staining with Alcian blue (Figure 5A). When Alcian blue was extracted from the stained cultures, only TGF-β3 supplementation led to increased proteoglycan deposition when compared with the control cells and those cultured with Link N (Figure 5B). Thus, Link N alone does not directly stimulate chondrogenesis in a manner analogous to TGF-β, but rather enhances ongoing chondrogenesis.

**Figure 6:**
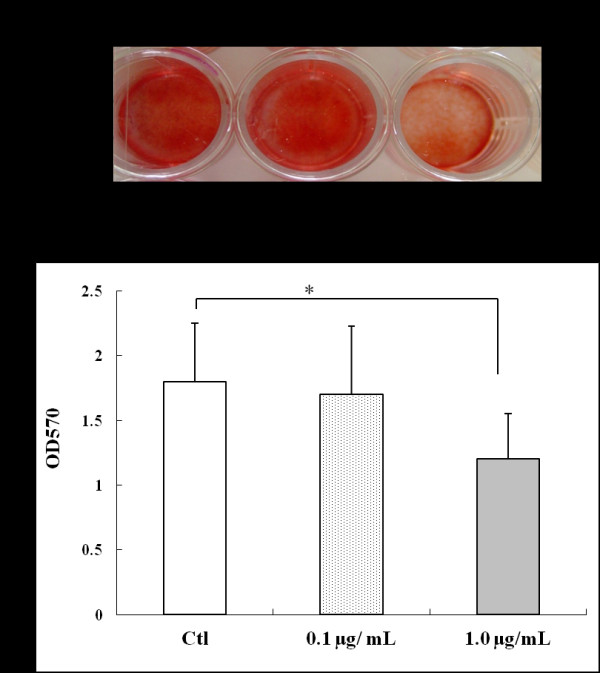
**Effect of Link N on mineral deposition by mesenchymal stem cells (MSCs) in osteogenic differentiation medium**. **(A) **Alizarin red staining of mineral deposition in the extracellular matrix after MSCs were cultured in osteogenic differentiation medium without (control) or with Link N for 21 days. **(B) **The absorbance value of solubilized alizarin red measured at 620 nm. The results are shown as mean ± SD of three independent experiments with MSCs from three different donors. *P < 0.05 versus control.

After MSCs were cultured in osteogenic differentiation medium without or with 0.1 or 1.0 μg/ml Link N for 21 days, mineral deposition in the accumulated ECM was visualized by staining with alizarin red S (Figure 6A). With the higher concentration of Link N, a decrease appeared to exist in the extent of matrix mineralization. When alizarin red S was extracted from the matrix, mineral deposition in the presence of 1.0 μg/ml Link N was shown to be decreased significantly compared with control cells (Figure 6B).

**Figure 7:**
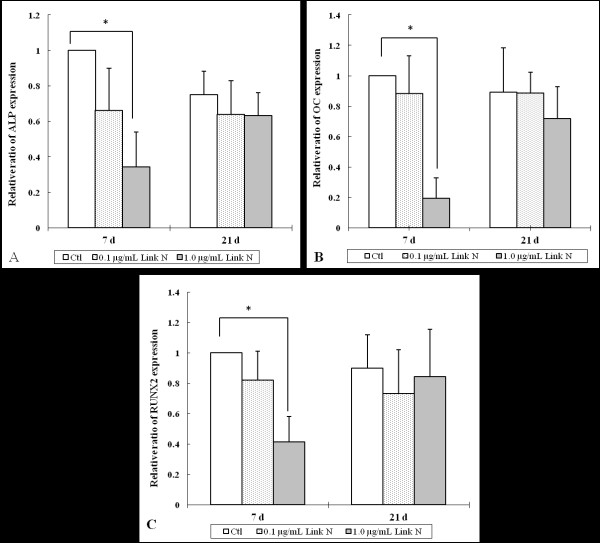
**The relative ratio of *ALP *(A), *OC *(B), and *RUNX2 *(C) gene expression in mesenchymal stem cells (MSCs) cultured in osteogenic differentiation medium without (control) or with 0.1 or 1.0 μg/ml Link N for 7 and 21 days**. The results are shown as mean ± SD of three independent experiments with MSCs from three different donors. *P < 0.05 versus control.

Alkaline phosphatase (*ALP*) and osteocalcin (*OC*) are important genes to define the osteogenic phenotype [43-45], and *RUNX2 *is an important transcription regulator of osteogenesis [45,46]. The effect of Link N on the expression of these genes was therefore assessed. After the cells were cultured in osteogenic differentiation medium with 1.0 μg/ml Link N for 7 days, *ALP *(*P *= 0.017), *OC *(*P *= 0.016), and *RUNX2 *(*P *= 0.019) expression decreased significantly compared with that in control cells (Figure 7A through C). No obvious effect was detected with 0.1 μg/ml Link N. At day 21, no significant difference of *ALP, OC*, and *RUNX2 *expression was observed compared with control cells for either 0.1 or 1.0 μg/ml Link N. Thus, at the higher concentration, Link N appeared to downregulate the osteogenic differentiation potential of the MSCs.

## Discussion

If Link N is to be used to stimulate disc repair in the presence of MSCs, it is essential that it does not interfere with chondrogenic differentiation and, ideally, promotes it. The results of the present work indicate that Link N can induce the expression of aggrecan and type II collagen, possibly through activation of *SOX9 *expression, and that the upregulation of aggrecan gene expression can increase proteoglycan production. Aggrecan and type II collagen are two important components of the extracellular matrix in IVD, and their degradation is associated with IVD degeneration [4,5,47]. Restoration of aggrecan and type II collagen levels within the disc ECM is therefore an essential requisite for disc repair. Thus Link N has the potential to be used together with MSCs in repairing degenerated IVDs.

It should be noted that although *in vitro *chondrogenesis is performed mostly in either pellet cultures or in a 3D environment, such as a hydrogel, the experiments presented here were not performed under these conditions. Monolayer culture was preferred, as it better mimics how stem cells will deposit on the disc ECM if they are to be injected *in vivo *for biologic repair. Future studies will be conducted to determine whether injection of Link N together with MSCs in an animal model of disc degeneration or into degenerated human discs in organ culture can promote ECM production.

A key consideration in using MSCs for repairing degenerated IVDs is not only the promotion of chondrogenesis, but also the prevention of hypertrophy and osteogenesis [48,49]. In the present work, such a process is indicated by decreased gene expression of type X collagen, a marker for hypertrophy, as well as ALP and OC, two important bone-matrix proteins synthesized by osteoblasts, and by decreased matrix mineralization through calcium deposition [50]. The ability of Link N to decrease the expression of ALP and OC may be through downregulating the transcription regulator RUNX2. Thus Link N can both favor chondrogenic differentiation of MSCs and retard their hypertrophic and osteogenic differentiation.

In the present study, it was not our intention to address directly the ability of Link N to promote chondrogenic, osteogenic, or disc-like differentiation. The goal of the work was to check that Link N does not negatively interfere with chondrogenesis, a differentiation pathway common to disc cells, which make matrix molecules common to all cartilages. Stem cell conversion to an NP-like phenotype rather than a hyaline cartilage is distinguished by the production of a gelatinous matrix with a proteoglycan-to-collagen ratio of approximately 28:1 [27]. When Link N is used *in vivo*, it is expected that growth factors such as TGF-β will always be present, and that these, coupled with the unique extracellular milieu, will guide the formation of either a disc-like or a cartilage-like matrix.

The influences of Link N on stimulating chondrogenesis and decreasing osteogenesis are similar to those reported with BMP and TGF-β [51,52]. However, one major advantage of Link N over a growth factor such as TGF-β for therapeutic use is the large saving in cost. For example, Link N costs $750 for 50 mg of the synthetic peptide, which is about 2 cents per microgram for supplementation. In contrast, TGF-β3 costs $30 per microgram. Link N therefore represents an economic therapeutic agent with potentially beneficial effects on disc repair when used either alone or in the presence of MSCs.

Previous studies have shown that Link N can stimulate the synthesis of proteoglycans and collagens in both articular cartilage and IVD [28-30]. The present data indicate that Link N and MSCs can synergize to stimulate chondrogenesis while suppressing osteogenesis. These are features needed not only for any agent designed to stimulate disc repair but also for any agent designed to stimulate articular cartilage repair. Therefore, in principle, disc and cartilage repair may be enhanced by stem cell supplementation in the presence of Link N to maximize extracellular matrix production. Such a beneficial effect does, however, assume that there is no nutrient deprivation that could impair cell metabolism. Although this could be a potential impediment to disc repair because of its avascular nature and the lack of a bathing nutrient fluid, it is less likely to be a detriment to articular cartilage repair where the tissue is bathed in synovial fluid and nutrient-diffusion distances are relatively short. Only future *in vivo *studies will be able to determine whether biologic repair of the degenerate disc by Link N and MSC supplementation could be a reality.

The present data indicate that Link N alone cannot directly promote MSC chondrogenesis. Thus, together with the other data, it appears that Link N enhances ongoing chondrogenesis rather than initiates it. Furthermore, Link N appears to be capable of reducing hypertrophic differentiation of chondrocytes. These results have implications in relation to regenerating a functional nucleus pulposus in the degenerated IVD or in repairing cartilage.

## Conclusions

In addition to promoting chondrogenesis and stimulating the gene expression of aggrecan and type II collagen, Link N also is able to downregulate osteogenesis and the gene expression oftype X collagen, alkaline phosphatase, and osteocalcin. Interestingly, these properties of Link N are similar to those reported previously for several growth factors and are features needed for any biologic agent designed to stimulate disc or cartilage repair. Therefore, in principle, Link N and MSC supplementation could have therapeutic value for the treatment of degenerative lesions in both disc and articular cartilage.

## Abbreviations

ACAN: aggrecan; ALP: alkaline phosphatase; COL2A1: collagen, type 2, alpha 1; DMMB: 1,9-dimethylmethylene blue; ECM: extracellular matrix; GAG: sulfated glycosaminoglycan; GAPDH: glyceraldehyde-3-phosphate dehydrogenase; IVD: intervertebral disc; MSC: mesenchymal stem cell; NP: nucleus pulposus; OC: osteocalcin; PCR: polymerase chain reaction; RUNX2: Runt-related transcription factor 2; SOX9: SRY (sex-determining region Y)-box 9.

## Competing interests

All the authors declare that they have no competing interests.

## Authors' contributions

JA supervised the research. HTW performed the experiments, data acquisition, and statistical analysis, as well as manuscript writing. AMA contributed to the analysis of osteogenesis by real-time RT-PCR. LH and PJR made substantial contributions to the study design and revised the manuscript. FM conceived and supervised the whole study and finished writing the manuscript. All authors read and approved the final manuscript.
